# The Multifaceted Role of Annexin A1 in Colorectal Cancer: From Molecular Mechanisms to Predictive and Prognostic Implications

**DOI:** 10.3390/medsci13040263

**Published:** 2025-11-10

**Authors:** Diana Lavinia Pricope, Adriana Grigoraș, Gabriel Mihail Dimofte, Cornelia Amalinei

**Affiliations:** 1 Department of Morphofunctional Sciences I, Grigore T. Popa University of Medicine and Pharmacy Iasi, 700115 Iasi, Romania; dianalavinia64@gmail.com (D.L.P.); cornelia.amalinei@umfiasi.ro (C.A.); 2 Department of Histopathology, Institute of Legal Medicine, 700455 Iasi, Romania; 3 Surgical Department, Grigore T. Popa University of Medicine and Pharmacy Iasi, 700115 Iasi, Romania; gdimofte@gmail.com; 4 2nd Clinic of Surgical Oncology, Regional Institute of Oncology, 700483 Iasi, Romania

**Keywords:** Annexin A1, colorectal cancer, rectal cancer, LARC, neoadjuvant chemotherapy, chemoresistance, prognostic marker, tumor microenvironment

## Abstract

Annexin A1 (ANXA1), a calcium-dependent phospholipid-binding protein, is considered a key modulator of cancer biology. Numerous pieces of evidence support its multifaceted involvement in tumor progression, metastatic dissemination, immune escape, and resistance to therapy in various malignancies, such as melanoma, along with liver, lung, and digestive tract tumors, including stomach and colorectal cancer (CRC). Although colon and rectal cancer (RC) exhibit overlapping characteristics, they are classified as separate clinical entities due to differences in tumor biology and therapy approaches. Moreover, locally advanced rectal cancer (LARC) raises clinical challenges due to variable treatment responses and its therapy resistance, preventing successful treatment and patients’ recovery. Considering ANXA1’s involvement in chemoresistance, further investigation is currently focused on ANXA1-targeted therapies. This review aims to update the knowledge on ANXA1, as a CRC predictive and prognostic biomarker, with involvement in therapy resistance, highlighting its significance in LARC patients. Through emerging evidence, our research provides valuable insights into the potential of ANXA1’s clinical utility and its prospective value as a target in chemoresistance approaches.

## 1. Introduction

Although colorectal cancer (CRC) is one of the most studied malignancies—with patients benefiting from a valuable screening program—it is still ranking among the top three cancers worldwide [1,2]. Regarding the location of this malignancy, rectal cancer (RC) accounted for approximately one-third of the cases, with an estimated 720,000 new diagnoses and 339,022 related deaths worldwide in 2020 [3,4]. Although it shares histological similarities with colon cancer, RC is recognized as a distinct clinical entity due to its key anatomical differences, e.g., blood supply, innervation, and pelvic anatomy characteristics, in addition to the different therapy strategies and different surgical management [5].

Locally advanced rectal cancer (LARC), which includes stage II (cT3–T4, N0) and stage III (cT1–T4, N1–N3) tumors, roughly comprises 5–10% of RC cases [2,6]. LARC treatment has a multimodal approach, with neoadjuvant chemoradiotherapy (nCRT), surgical resection, and, in selected cases, adjuvant chemotherapy [4,7,8]. However, LARC represents a biologically heterogeneous disease, characterized by tumor biology differences, showing multiple alterations of oncogenes and tumor suppressor genes [9,10]. This molecular diversity contributes to variable responses to nCRT, with a significant influence on patients’ survival. In medical practice, only a minority of patients (about 15–27%) achieve a complete pathological response (pCR), while 15–45% of patients show resistance to neoadjuvant therapy [10,11]. In agreement with these findings, pCR absence following nCRT in LARC patients is correlated with a poor prognosis, including an increased risk of local recurrences and distant metastases [12,13]. In this context, developing a system that enables an accurate identification of patients with colon cancer and RC, which may benefit from this therapeutic approach, is essential, avoiding unnecessary radiation exposure and preventing the surgical intervention-associated morbidity [12,13,14]. Thus, the validation of biomarkers that are able to guide therapeutic decision-making in LARC patients and in the prediction of tumor response to nCRT is currently a priority. Despite significant efforts in the identification of promising biomarker candidates, there are still challenges in their validation [15], without any current validated biomarker that can be used as a reliable predictive tool in routine clinical use [16].

Annexin A1 (ANXA1), the first identified member of the annexin protein family, is characterized by its ability to bind phospholipids using a calcium-regulated mechanism [17,18,19,20]. ANXA1 plays a pivotal role in tumor progression and in different cellular processes, e.g., cell proliferation, differentiation, migration, and apoptosis [21,22]. Increasing evidence highlights its involvement in the pathogenesis of numerous malignancies, such as skin squamous cell carcinoma, prostate, liver, stomach, laryngeal, breast, or bile duct cancer, and CRC [17,23]. Moreover, several studies support that ANXA1 is frequently linked to advanced stage, aggressive tumor behavior, and poor outcome, being consequently regarded as an independent prognostic marker of a patient’s survival [17,24].

Considering its functional roles, ANXA1 contributes to chemoresistance, mainly to 5-fluorouracil (5-FU), a fundamental chemotherapeutic agent used in the management of both RC and colon cancers [25,26]. In addition to its involvement in chemoresistance, ANXA1 has also been associated with resistance to nCRT in RC, suggesting its potential value as a biomarker in the prediction of the clinical outcome [27]. Given that drug resistance represents a major barrier in successful treatment outcomes, further investigation of the underlying mechanisms of nCRT resistance is needed [25,27], with a special focus on the elucidation of ANXA1’s role in LARC [28].

Despite the relatively limited literature data, ANXA1 may possess some underexplored functions that could significantly contribute to the identification of the resistance mechanisms, leading to a better outcome for LARC patients undergoing neoadjuvant treatment. In this context, our review aims to provide an updated overview of ANXA1 roles in CRC progression, with a specific focus on LARC, in addition to its potential role as a predictive and prognostic biomarker in the settings of nCRT.

## 2. The Annexin Protein Superfamily

The annexin family represents a superfamily of calcium-regulated phospholipid-binding proteins, consisting of 13 members, with specific structural features and variable biological roles [27,29]. A notable characteristic of these proteins is their tissue-specific expression pattern [20]. Thus, ANXA1 and Annexin A2 (ANXA2) are highly expressed in endothelial cells [30], while Annexin A3 (ANXA3) and Annexin A11 (ANXA11) are mainly detected in neutrophils and in peripheral blood cells [31]. Additionally, Annexin A4 (ANXA4) is localized in vestibular and cochlear hair cells [32], while Annexin A5 (ANXA5) is involved in the activation of T cells [32,33].

Annexins’ tissue-specific expression is also noted in a spectrum of tumors, including lung, liver, ovarian, and breast cancers [24,27,34]. Related to their involvement in different malignancies, ANXA2 may facilitate angiogenesis and metastatic spread by promoting the production of fibrinolytic enzymes in breast cancer [20,32]. Conversely, ANXA6 seems to be downregulated in gastric cancer while showing the ability to inhibit tumor progression by Rat Sarcoma/Mitogen-Activated Protein Kinase (Ras/MAPK) signaling pathway inactivation [35,36,37]. In addition to their roles in cellular signaling and membrane dynamics, several annexins may also exhibit anticoagulant and anti-inflammatory roles, in addition to the mediation of cell-to-extracellular matrix interactions [20,31,37].

Annexins have an elaborate structure, characterized by a compact, predominantly alpha-helical core domain [20,22,31,37,38], functioning as a calcium-dependent membrane-binding module by regulated interactions with phospholipid membranes [22,39]. Notably, specific annexin family members, such as ANXA1, ANXA2, and ANXA5, have variable structures, including the capacity to produce Ca^2+^-dependent membrane-bound trimers [22]. Moreover, their unique N-terminal domains undergo post-translational changes and mediate interactions with other proteins, supporting their specific subcellular localization and biological roles [30].

## 3. ANXA1’s General Structure and Function

ANXA1, the first identified member of the annexin family, is expressed in a variety of cell types, including endothelium and other epithelial cells, as well as lymphocytes and leukocytes [22,30]. ANXA1 is a 346-amino-acid protein, comprising two main structural regions: the N-terminal tail and the conserved C-terminal core domain [40,41]. The C-terminal domain contains calcium- and membrane-binding sites that regulate the conformational changes, leading to N-terminal tail exposure [22]. This N-terminal region plays a crucial role in the protein’s biological activity by interaction with various ligands, resulting in post-translational changes, such as acetylation, phosphorylation, and glycosylation [22]. These changes lead to ANXA1 involvement in a wide range of physiological and pathological processes, including cell adhesion, cytoskeleton rearrangement, apoptosis, angiogenesis, immune modulation, inflammation, and differentiation, as well as cancer cell proliferation, invasion, and metastasis [30,42,43,44].

The C-terminal domain of ANXA1 consists of four homologous repeats, each of approximately 70 amino acids in length. Every repeat is made up of five tightly packed α-helices, which collectively fold into a slightly curved, disc-shaped structure [45]. The four repeats are organized in a specific sequential manner (I, IV, II, and III), resulting in a stable, hydrolysis-resistant compact form via hydrophobic interactions [42,45]. This core region includes several calcium-binding sites that facilitate an ANXA1 calcium-dependent attachment to the cellular phospholipid membranes. ANXA1 domains 2 and 3 play a key role in the mediation of the interaction with membrane phospholipids by a calcium-dependent binding mechanism [38,42,45]. In contrast, the N-terminal region, located far from the membrane, is more susceptible to interactions with variable intracellular components and provides functional specificity to all annexin family members [46,47].

Structural studies using crystallography revealed that the N-terminal domain consists of two alpha-helices, positioned at approximately a 60-degree angle [38,48]. This region remains closely associated with domain 3—if Ca^2+^ ions are absent—while calcium binding leads to a conformational change of ANXA1 that exposes the N-terminal domain, allowing the C-terminal region to bind phospholipids [49,50]. This structural rearrangement enables the amphipathic helix of the N-terminal region to engage with an additional phospholipid-binding site, thereby promoting membrane aggregation or interactions with different cellular mediators [38,50].

Regarding its cellular localization, ANXA1 is found in the plasma membrane, cytoplasm, or nucleus, localizations that support its involvement in key cellular processes, such as proliferation, survival, differentiation, migration, and apoptosis [38,47,51].

ANXA1 exhibits both RNA- and DNA-binding abilities in the nucleus, attributed to specific sequence motifs and structural domains [38,42]. These interactions are regulated by two divalent cations, namely Mg^2+^ and Ca^2+^ [38,42]. Moreover, ANXA1 possesses helicase-like activity [52,53]. The presence of Mg^2+^ ions and ATP allows the nuclear ANXA1 binding to DNA, promoting strand separation by ATP hydrolysis [38,54]. Conversely, in a Ca^2+^-rich milieu, it facilitates the annealing of complementary single-stranded DNA segments [38,54].

ANXA1 is also widely distributed in different cytoplasmic compartments, including the inner part of the plasma membrane and vesicular structures, such as phagosomes and endosomes, and, occasionally, in the endoplasmic reticulum [38,55]. Cytoplasmic ANXA1 plays a key role in the modulation of the inflammatory responses by inhibition of phospholipase A2, thereby reducing the release of arachidonic acid and its downstream inflammatory metabolites [56]. By its involvement in lipid metabolism pathways, ANXA1 facilitates leukocyte transmigration through the endothelium and contributes to tissue repair processes [57]. Furthermore, ANXA1 downregulates the expression of cyclooxygenase-2 (COX-2), leading to suppressed prostaglandin synthesis and reduced inducible nitric oxide synthase (iNOS) levels, reinforcing its anti-inflammatory role, with a significant contribution to cancer progression modulation [38,58].

## 4. ANXA1’s Role in Cancer

The aim of several recent studies has been the assessment of ANXA1’s therapeutic value in cancer [22,30,38]. Emerging evidence shows that ANXA1 plays critical roles in tumorigenesis, including the modulation of oncogene activation, suppression of tumor suppressor genes, and promotion of cellular invasion and proliferation [38,59,60]. However, ANXA1 exhibits both tumor-promoting and tumor-suppressing properties, depending on the cancer type and stage [61]. Strong ANXA1 expression has been reported in various malignancies, such as melanoma [62], glioma [63], breast cancer [64], lung cancer [65], hepatocellular carcinoma [66], and CRC [67,68], including LARC [27], being mostly associated with a poor prognosis along with a reduced overall survival (OS) and disease-free survival (DFS) [65,66]. Conversely, diminished ANXA1 expression has been related to a poor outcome, with reduced survival rates and higher relapse incidence [38,69] in other types of cancers, e.g., thyroid carcinoma [69] and head and neck squamous cell carcinoma [70,71].

Moreover, ANXA1 plays a central role in the modulation of the immune response in cancer. In this regard, ANXA1-increased expression in triple-negative breast cancer (TNBC) promotes Treg cell-mediated immune suppressive activity [41]. A strong association between high ANXA1 expression and a poorly differentiated tumor grade was detected and associated with decreased survival rates [64,72]. ANXA1 overexpression was also related not only to TNBC but also to lymph node metastasis [73]. Supporting these findings, ANXA1 has a critical role in breast oncogenesis, particularly by stimulation of the epithelial–mesenchymal transition (EMT), thereby promoting tumor migration and metastasis [38]. Additionally, ANXA1 exhibits pro-angiogenic effects in breast cancer, notably through the activation of the nuclear factor-kappa B (NF-κB) signaling pathway [74].

ANXA1 also plays a significant role in the progression of other types of cancer, including gastric cancer, with its overexpression being associated with peritoneal metastasis and advanced tumor stages [61,75]. In addition, ANXA1 contributes to tumor cell migration and dissemination by autophagy inhibition in nasopharyngeal carcinoma [76]. Similarly, high ANXA1 expression has been associated with increased invasion and migration, as well as the development of bone metastases in bronchopulmonary cancers [77].

Numerous ANXA1 oncogenic functions are also mediated by FPR1 (formyl peptide receptor 1)-dependent signaling pathway activation [38]. FPR1 activation promotes multiple cellular processes, such as chemotaxis, proliferation, and angiogenesis, in glioblastoma multiform [78,79]. In this respect, ligands released by necrotic glioblastoma cells, along with ANXA1, can mediate FPR1 activation [80]. Additionally, FPR1 may contribute to gliomas and neuroblastoma progression by transactivation of the epidermal growth factor receptor (EGFR) [79,81]. FPR1 can also be activated in TNBC via an autocrine mechanism by ANXA1’s N-terminal peptide that is secreted by the MDA-MB-231 cell line [38,82]. Thereby, the autocrine AnxA1/FPR1 signaling promotes malignant abilities, migration, and proliferation of cancer cells [73]. Furthermore, FPR1 overexpression has been registered in gastric cancer in association with tumor progression and an unfavorable prognosis [83].

Taken together, the current data show that ANXA1 plays a major role in several oncogenic processes, such as angiogenesis, DNA damage response, tumor cell proliferation, along with cancer cell invasion and migration [38]. In addition, ANXA1 is associated with metastatic dissemination and immune modulation, resulting in immune suppression [84,85]. A summary of ANXA1’s roles in different types of cancer and identified in different study models concerning tumorigenesis, proliferation, the immune response, tumor growth, invasion, EMT, metastasis, and tumor grading, along with their significance in prognosis, therapy response, OS, and PFS [41,62,85,86,87,88,89,90,91,92,93], is illustrated in Table 1.

## 5. ANXA1 Role in CRC

### 5.1. ANXA1 as a Biomarker in CRC Progression

Added to its variable roles in different malignancies, ANXA1 is involved in CRC progression, representing a potential biomarker [94]. Recent evidences show that ANXA1 is upregulated in CRC, exhibiting a strong correlation with an advanced TNM stage, lymph node involvement, and tumor invasion [94]. Thus, positive ANXA1 immunoexpression has been observed in 76–86.67% of CRC in TNM stages III–IV, compared to 24–64% in patients with TNM stages I–II [17,94]. Additionally, ANXA1 expression may be correlated with lymphatic invasion (*p* = 0.011), venous invasion (*p* = 0.023), and lymph node metastasis (*p* = 0.042) in CRC patients [17], suggesting its potential role in tumor invasion and lymph node metastasis.

In the context of the CRC Consensus Molecular Subtypes (CMS) classification, a strong expression of focal adhesion proteins, such as ANXA1, ANXA6, vimentin, nexilin, filamin, actinin-α1, and caveolin-1, has been associated with CMS4 CRC cells’ resistance to chemotherapy [95].

Furthermore, a significant association between ANXA1 overexpression and increased carcinoembryonic antigen (CEA) levels demonstrates its involvement in CRC progression [21]. The comparative analysis of tumor tissues and adjacent margins has also shown a stronger ANXA1 expression in cancer samples (*p* = 0.0001), in correlation with elevated CEA serum levels (*p* = 0.004), suggesting that ANXA1 may play a key promoter role in CRC aggressiveness and disease progression [21]. Moreover, ANXA1 expression is significantly increased in sentinel lymph node micrometastases compared to non-metastatic lymph nodes (*p* < 0.01), suggesting its involvement in the early phases of CRC metastasis [96].

Considering that traditional serum biomarkers such as CEA and CA19-9 show limited sensitivity and specificity, the search for new CRC markers, such as ANXA1, SCAT1, SCAT2, and SCAT8, has been initiated [97,98,99]. However, ANXA1 serum values are variable in different cancer types, including CRC. For instance, reduced ANXA1 serum values have been reported in patients with oral and oesophageal squamous cell carcinoma compared to healthy controls [19,100]. In contrast, an increased ANXA1 serum value has been reported in melanoma and lung cancer patients [77,101]. Moreover, ANXA1 serum values are significantly higher in lung cancer patients with brain metastases compared to lung cancer patients without brain metastases, suggesting its potential value in metastasis detection [102]. Regarding CRC, an increased ANXA1 serum level has been detected in a group of 95 CRC patients compared with a healthy individuals group (*p* < 0.05) [94]. However, a limited number of studies show that CRC patients exhibit a reduced ANXA1 serum value, accompanied by increased inflammatory markers, including IL-6 and sPLA2 [103]. Although these data suggest an association between a low ANXA1 value and systemic inflammation, further clinical studies are needed to assess its prognostic and clinical predictive value. Additionally, standardized assays are required to implement ANXA1 serum detection in clinical practice, considering its complex dual role in tumor progression and metastasis.

Added to ANXA1, long non-coding RNAs (lncRNAs) SCAT1, SCAT2, and SCAT8 may represent other potential novel biomarkers for early CRC detection [97]. In this respect, significant SCAT1, SCAT2, and SCAT8 overexpression was detected in CRC tissues compared with adjacent non-cancerous tissues (*p* = 0.001, *p* < 0.0001, and *p* < 0.0001, respectively), in a recent study [97]. Statistical analyses demonstrated that SCAT2 and SCAT8 may serve as promising CRC diagnostic biomarkers, whereas SCAT1 exhibits only a moderate diagnostic potential value [97]. Taken together, these findings suggest that SCAT1, SCAT2, and SCAT8 may act as new oncogenic drivers and prognostic markers in CRC, potentially contributing to carcinogenesis and tumor progression.

A recent study using HCT116 and SW620 CRC cell lines demonstrated that ANXA1 exhibits both diagnostic and prognostic biomarker potential, while also emphasizing its variable expression in different cellular models [68]. Thus, the regulatory role of cell proliferation may be associated with high ANXA1 expression in HCT116 CRC cells or with low ANXA1 expression in SW620 CRC cells [68]. However, this regulatory effect seems to be mediated by different mechanisms, with the modulation of in vitro cell cycle progression and by in vivo tumor growth promotion [68]. Moreover, another mechanism used by ANXA1 may be that of inhibition of honokiol-induced autophagic tumor cell death by mitochondrial reactive oxygen species (ROS) stabilization in CRC [68].

The literature data suggest that ANXA1 may possess a role in tumor resistance to nCRT in LARC patients, albeit relatively limited direct investigations of an ANXA1-nCRT response have been performed [27]. However, ANXA1 overexpression is significantly correlated with more advanced stages, both before and after treatment, as well as more extensive lymph node involvement in RC patients undergoing nCRT [27], with these associations suggesting unfavorable clinical outcomes and poor therapeutic responses. For example, a study involving 172 LARC patients found that high levels of ANXA1 were linked to advanced pre-therapeutic T stages (T3 and T4, *p* = 0.022), advanced pre- and post-therapeutic nodal status (N1/N2, *p* = 0.004, and *p* = 0.001, respectively), and a low tumor regression grade following nCRT (*p* = 0.009) [27]. Additionally, this study identified ANXA1 as a prognostic marker for metastasis-free survival (MFS) (*p* = 0.0004), local recurrence-free survival (LRFS) (*p* = 0.0001), and disease-specific survival (DSS) (*p* < 0.0001), highlighting its association with a poor overall prognosis [27]. Similarly, strong ANXA1 expression may be considered a prognostic marker in colon cancer, given its significant association with tumor growth and metastasis [17].

Overall, these findings strongly support the ANXA1 value as a predictive and prognostic biomarker in CRC and RC, given its association with nCRT poor response, advanced disease stages, and aggressive tumor features, with some specific functional correlations for each location.

### 5.2. Functional Mechanisms of ANXA1 in CRC Progression

#### 5.2.1. ANXA1 in CRC Apoptosis

Apoptosis, or programmed cell death, plays a significant role in the regulation of tumor development and in chemotherapy and radiotherapy effectiveness [104]. Considering that ANXA1 is correlated to apoptosis regulation, it may be a potential target for therapeutic strategies that are aimed at modulating CRC cell death pathways [22].

**Figure 1:**
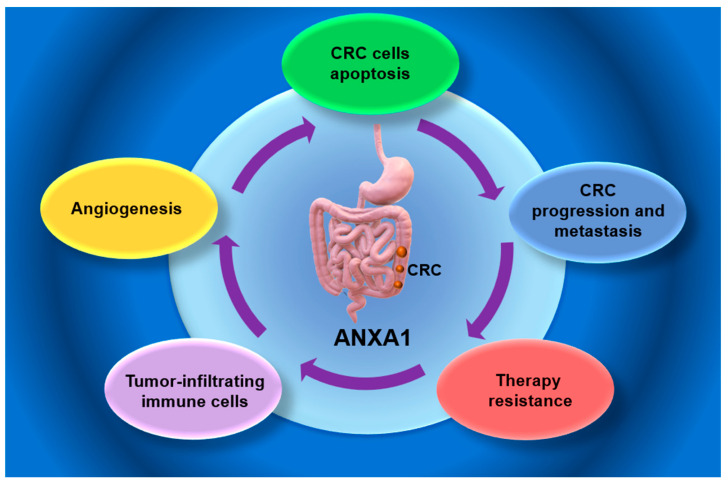
Potential role of Annexin A1 in colorectal cancer (CRC). ANXA1—Annexin 1; CRC—colorectal cancer.

Several studies have demonstrated that all members of the annexin family, including ANXA1, can exhibit both pro-apoptotic and anti-apoptotic functions [20,105,106]. For example, increased ANXA1 expression in bronchoalveolar carcinoma and histiocytic lymphoma has been associated with a transient increase in intracellular Ca^2+^ and caspase-3 activation [107]. Conversely, reduced ANXA1 expression has been associated with enhanced apoptosis in glioma cells [26]. Furthermore, inhibition of ANXA1 expression promotes apoptotic cell death [87,108,109]. Moreover, ANXA1 overexpression enhances CRC cell survival under 5-FU treatment, while its knockout decreases viability and restores apoptosis sensitivity [25] (Figure 1).

The NF-κB signaling pathway represents another key regulator of inflammation, CRC cell survival, apoptosis, and therapeutic resistance [110]. Activation of the NF-κB pathway leads to apoptosis inhibition by the upregulation of several anti-apoptotic mediators, including BFL-1/A1, Bcl-xL, apoptosis inhibitors, and c-FLIP, a caspase-8-like regulator that interferes with death receptor-mediated signaling [111,112]. Conversely, NF-κB inhibition in colon cancer and RC may re-establish apoptotic sensitivity [112]. Thus, quinacrine may suppress NF-κB activity, enhancing TRAIL-induced cytotoxicity in RKO and HT-29 cell lines [110,112]. Moreover, ANXA1 has been identified as an upstream activator of NF-κB in CRC chemotherapy [25]. Thus, ANXA1 is upregulated in 5FU-resistant CRC, contributing to treatment resistance both by NF-κB activation and by the promotion of the transcription of survival-related genes, such as XIAP and BCL-2, associated with the concomitant inhibition of pro-apoptotic factors [25]. Moreover, ANXA1 facilitates the nuclear translocation of the p65 subunit of NF-kB, followed by the proliferation and anti-apoptotic signaling increase [105]. Additionally, CRC cells are able to evade apoptosis in response to cytotoxic stressors, including hypoxia and 5FU exposure, via the ANXA1–NF-κB axis [25,105]. Consequently, targeting NF-κB signaling could counteract this effect, highlighting its potential use in the therapeutic strategy of CRC chemosensitivity enhancement [25,105].

Considering their role in apoptosis regulation, the BCL-2 family of proteins may also provide potential CRC therapeutic targets [113]. A reduction in anti-apoptotic proteins, such as Bcl-2, and an increase in pro-apoptotic proteins, like Bcl-2-associated X protein (Bax), are associated with ANXA1 suppression [68,114,115]. However, the expression of BAX-interacting factor-1 (BIF-1)—a protein that supports Bax-mediated apoptosis—is significantly decreased in CRC tissues [116], resulting in impaired Bax activity, defective apoptosis, and reduced therapy response [116].

Taken together, in both colon cancer and RC, high ANXA1 expression is associated with therapy resistance and apoptosis inhibition, while its suppression promotes cell death by reducing the anti-apoptotic markers, i.e., Bcl-2, and by increasing pro-apoptotic proteins, i.e., Bax.

#### 5.2.2. ANXA1 Involvement in CRC Tumor Microenvironment (TME)

The tumor microenvironment (TME), a complex ecosystem composed of both malignant and non-malignant cells, as well as their secreted products, is involved in CRC metastasis [117,118,119]. TME includes stromal fibroblasts, immune cell populations, extracellular matrix components, signaling molecules, and blood vessels [120,121], with a significant role in cancer progression and dissemination by the facilitation of tumor immune escape [120].

TME significantly influences the nCRT response in RC [122,123]. In this regard, intratumoral budding (ITB) and tumor-infiltrating lymphocytes (TILs) have been identified as predictive markers of an nCRT response, while tumor-associated macrophages (TAMs) provide metabolic support, enhancing cancer cells’ resistance to therapy [122,124]. Furthermore, dendritic cells (DCs), as antigen-presenting cells, contribute to anti-tumor immunity, whereas cancer-associated fibroblasts release factors that change the extracellular matrix to support cancer stemness [123,125].

ANXA1 is involved in TME regulation, with an impact on tumor proliferation, metastasis, immune evasion, and therapy resistance [126]. Both stromal and tumor ANXA1-expressing cells contribute to TME communication by autocrine, paracrine, and juxtacrine signaling pathways, stimulating tumor cell proliferation, disease progression, invasion, metastasis, and resistance to therapy [23,126,127]. These findings support ANXA1’s central role in TME modulation and point out ANXA1-mediated signaling as a promising therapeutic target [23].

The immune system represents a vital TME component, playing a crucial role in cancer progression, mainly by immune evasion [126]. As a result, targeting the TME immune landscape by immunotherapy can lead to cancer therapy outcome improvement [126]. ANXA1, together with other related genes, forms a regulatory network involved in the cellular response to chemokines, enhancing chemotaxis and facilitating the recruitment of variable immune cells, such as neutrophils, macrophages, and DCs [126,128]. Additionally, ANXA1 expression is associated with M2-polarized macrophages’ surface markers, such as CD163, MS4A, and USIG4, which are usually related to immunosuppression in CRC TME [126,128].

While the prognostic value of ANXA1 has been well-documented in various cancers, its immune modulation role in CRC is still underexplored [34,128]. Beyond its prognostic and predictive value, ANXA1 may actively regulate the specific interactions within CRC TME compartments [23,128], demonstrated by the association between strong ANXA1 expression and tumor-infiltrating immune cells, leading to poor patient survival [128].

Data from The Cancer Genome Atlas (TCGA) and the Gene Expression Omnibus (GEO) allow a comprehensive bioinformatics analysis, while the Tumor Immune Estimation Resource (TIMER) provides tools to explore the relationship between ANXA1 expression and immune cell infiltration in CRC tissues [128]. Their data suggest that ANXA1 plays a dual role, contributing to both immune cell infiltration and metastasis, potentially due to its function as an inflammatory mediator and proliferation regulator [128]. Moreover, significant associations between ANXA1 expression and several CRC TME immune cell types, including CD4^+^ and CD8^+^T cells, B cells, neutrophils, macrophages, and DCs, were detected [128]. Interestingly, ANXA1 expression exhibits a moderate to strong positive association with neutrophils, macrophages, and DCs and a weak to moderate association with B cells and T cell subsets (CD4^+^ and CD8^+^) [128], supporting ANXA1’s value as a potential biomarker for CRC immune landscape characterization. These findings were supplemented by the analysis of the correlations between ANXA1 mRNA levels and markers of different immune cell types [129], revealing a significant association between ANXA1 expression and the detection of myeloid DCs and monocytes in the TME of several cancer types, including renal cancer, breast cancer, and CRC [129]. These results suggest that ANXA1 may play an important role in the recruitment of myeloid-derived cells, including dendritic cell precursors, into the tumor tissues [129].

##### Neutrophils

ANXA1 is significantly expressed in the cells of the innate immune system, including neutrophils, macrophages, monocytes, and mast cells, while its expression is relatively low in adaptive immune cells, such as B and T lymphocytes [23]. Considering its cellular roles, ANXA1 inhibits neutrophil migration and adhesion, promotes the clearance of apoptotic neutrophils by macrophages, and modulates macrophage differentiation within the context of the innate immune response [23,130].

The tumor-associated neutrophils (TANs) are able to differentiate into distinct functional phenotypes depending on TME [23]. Thus, neutrophils can adopt the N1 phenotype, which supports anti-tumor immunity by counteracting transforming growth factor-β (TGF-β) signaling [131] or, under the influence of TGF-β, neutrophils may switch into the N2 phenotype, which is characterized by immunosuppressive abilities that facilitate tumor progression [131].

Although recognized for its anti-inflammatory role, ANXA1 may also exhibit a pro-inflammatory function, depending on the interacting ligands [132]. In this respect, ANXA1 acts on immune cells by its interaction with formyl peptide receptors (FPRs), mainly FPR2/ALX [126]. Via the FPR pathway activation, neutrophil-derived ANXA1 can stimulate cancer cell invasion and metastasis [101], including in CRC [133]. Conversely, exogenous ANXA1 can suppress neutrophil activation by binding to FPR2/ALX, highlighting its dual regulatory role in inflammation within the TME milieu [126].

The dual role of neutrophils in cancer is recognized [117], with either anti-tumor effects, such as growth of tumor-associated microbiota limitations and metastasis suppression in experimental CRC [134], or tumor progression and metastasis support via the CXCL1/CXCR2 chemokine axis activation [117,135]. Moreover, increased TME neutrophil infiltration and the neutrophil-to-lymphocyte ratio have also been associated with nCRT resistance and a poor prognosis in LARC patients [136].

##### Macrophages

Macrophages play a central role in CRC progression and resistance to therapy [117,137]. According to their two main subtypes [117,138], M1 macrophages are recognized for their anti-tumor effects by immune responses stimulation [139], whereas M2 macrophages support tumor growth and invasion stimulation by the release of matrix metalloproteinases, angiogenesis promotion by vascular endothelial growth factor A (VEGF-A), and tumor cell survival support by epidermal growth factor (EGF) and fibroblast growth factor-1 (FGF-1) production [117]. Additionally, M2 macrophages may suppress anti-tumor immune responses, including in LARC patients, by their ability to produce different cytokines, such as IL-6, IL-10, and TGF-β1 [117,140].

ANXA1’s N-terminal domain facilitates the transformation of macrophages into the pro-neoplastic M2 phenotype [38], while the interaction between ANXA1 and FPR2 enhances M2 polarization and infiltration, contributing to an immunosuppressive TME [23]. In this context, a bioinformatics analysis using CIBERSORT and TIMER platforms, using TCGA CRC samples, has confirmed a positive correlation between ANXA1 expression and M2 macrophage infiltration [128]. Moreover, ANXA1 expression exhibits a weak correlation with M1 macrophages gene markers and a moderate to strong correlation with markers associated with M2 macrophages [128], suggesting that it may play a role in TAMs polarization, favoring the M2 phenotype transition, which is usually associated with a poor prognosis in different cancer types, including CRC [128,141]. Consequently, high ANXA1 expression is associated with an immunosuppressive, macrophage-dense TME that supports CRC progression [141].

##### T Lymphocytes

TILs, comprising various immune cell subsets, such as CD4^+^ and CD8^+^T cells, natural killer (NK), and B cells, are key TME components in different types of cancers, including colon cancer and RC [117,140,142]. Among these types, T cell subpopulations are significant, considering their roles in the modulation of the immune response to tumors. In this frame, CD3^+^ and CD8^+^T cells have high infiltration, added to CD4^+^FoxP3^+^Tregs’ low infiltration, which has been observed in LARC patients after nCRT, suggesting that therapy may induce an immune-active TME [140].

In addition, ANXA1 involvement in the differentiation of T helper 1 (TH1) cells [23,130] and the infiltration of Th2 cells may be associated with pancreatic cancer’s poor prognosis [143]. Furthermore, ANXA1 is involved in Tregs control, impairing the induction of antigen-specific cytotoxic T cell responses [38]. These mechanisms allow ANXA1 to support an immunosuppressive TME that stimulates tumor progression and metastatic spread [38].

Considering that the density of tumor-infiltrating T lymphocytes is closely associated with disease progression, with a critical role of adaptive immunity within CRC TME [144,145], bioinformatics analyses using CIBERSORT and TIMER on CRC datasets have shown a significant correlation between ANXA1 expression and the infiltration of CD4^+^ and CD8^+^ T cells, suggesting a high lymphocyte density in tumors with high ANXA1 expression [128].

The immunosuppressive effects of ANXA1 in CRC are also supported by its interaction with EGFR, a major signaling molecule involved in tumor development and therapeutic resistance [38,146]. While EGFR signaling is a central target in metastatic CRC therapy [146,147], ANXA1 may enhance both EGFR activation and stabilization, thereby contributing to cancer immune evasion via the ANXA1/EGFR interaction [42]. Additionally, ANXA1 facilitates the activation of STAT3, a transcription factor that plays a key role in CRC initiation and progression [148,149].

Collectively, these findings highlight ANXA1’s critical role in the promotion of an immunosuppressive CRC microenvironment through its interactions with EGFR and STAT3 signaling pathways [38]. Thus, targeting these ANXA1-mediated pathways represents a promising future CRC therapeutic strategy.

##### Dendritic Cells

DCs (derived from myeloid progenitor cells)—key components of the adaptive immune system—play a crucial role in T cell-regulated anti-tumor responses [117]. A strong association between ANXA1 expression and the infiltration of myeloid DCs and monocytes in cancers characterized by active immunosurveillance, such as CRC, breast, and renal carcinomas, has been detected [129]. Accordingly, tumors exhibiting a low ANXA1 expression contain more cytotoxic T lymphocytes and DCs, suggesting that reduced ANXA1 expression may contribute to immune evasion mechanisms [23,129]. This finding underscores ANXA1’s role in the recruitment of myeloid cells, including DCs, into TME. Furthermore, ANXA1 immunomodulatory effects on DCs are variable according to its structural domains and the release modality [23]. Thus, ANXA1 can enhance the expression of anti-inflammatory mediators, supporting immune tolerance, if its core domain interacts with DC surface receptors [23]. Conversely, the involvement of DC-expressed FPRs by the ANXA1 N-terminal region has been associated with an increased immune activation [23]. Moreover, the impairment of DCs activation, influenced by ANXA1 signaling, can suppress CD8^+^T cells recruitment, thereby enabling tumor progression [29]. Within CRC TME, ANXA1 may regulate DCs’ functional state by inducing cytotoxic mediators’ production, while concurrently diminishing interferon-gamma secretion by CD4^+^T cells [29].

Additionally, ANXA1 expression shows significant correlations with markers of TME Tregs and DCs [128] (Table 2). These findings support the potential involvement of ANXA1 in immune cell behavior regulation, supporting tumor immune evasion and metastasis [150]. Considering that DCs contribute to metastatic spread by CD8^+^T cell responses and Treg activity regulation, ANXA1 can be considered a prognostic indicator of CRC metastasis and a possible target for immunomodulatory therapies [128,150].

#### 5.2.3. ANXA 1 Role in CRC Angiogenesis

Angiogenesis is essential for cancer development and progression, considering that new blood vessels provide the vascular structure required for tumor growth in both primary and metastatic sites [151]. Added to its well-established roles in cytokine regulation and inflammation, ANXA1 significantly contributes to tumor angiogenesis [23,24,152]. Regardless of the absence of vascular anomalies, ANXA1 lacks the ability to impair tumor-induced neovascularization, leading to tumor growth and metastasis suppression in ANXA1-knockout mice [23,153].

Although the exact mechanisms of ANXA1 involvement in angiogenesis are not yet fully understood, its expression is increased in various malignancies, including CRC, melanoma, and lung cancer [23,154]. ANXA1 functions in an autocrine manner by endothelial interaction with FPRs, enhancing the release of VEGF [151,155,156], a major pro-angiogenic factor that is involved in CRC progression and dissemination [151,157]. VEGF-A promotes angiogenic signaling by triggering ANXA1 phosphorylation via the p38/MAPKAP kinase-2/LIMK1 pathway, facilitating endothelial cell angiogenesis [23]. Thus, VEGF-A high expression is associated with a poor prognosis in LARC patients [158].

Taken together, these findings support ANXA1’s central role in the angiogenesis process and, thus, targeting ANXA1 represents a promising therapeutic strategy in vascularization inhibition and, consequently, in cancer progression inhibition [23].

## 6. ANXA1 in CRC Chemotherapy and Radiotherapy Resistance

### 6.1. CRC Treatments: Past, Present, and Future

Over the last four decades, cancer treatment has significantly changed [159]. Advances in surgical techniques have been complemented by the development of radiotherapy and mainly 5-FU-based chemotherapy as a pivotal strategy in CRC management [160]. Currently, the standard CRC management involves surgical resection, followed by adjuvant chemotherapy, which is typically based on 5-FU combined with platinum compounds [161]. Since the early 2000s, the first-line therapy for advanced CRC has consisted of a combination regimen, incorporating 5-FU/leucovorin with either oxaliplatin or irinotecan [162].

Moreover, targeted agents have expanded the therapeutic landscape, including the approval of anti-EGFR monoclonal antibodies (cetuximab and panitumumab) and anti-VEGF therapies (bevacizumab and aflibercept) [162]. Recent advances in immuno-oncology have also demonstrated that immune checkpoint inhibitors (ICIs) provide clinical benefit in patients with deficient mismatch repair or high microsatellite instability (dMMR/MSI-H) tumors [163]. Despite these promising outcomes, only about 15% of CRC patients fall within this responsive subgroup [163], with treatment resistance and disease progression being the major challenges of current therapeutic options [162,164]. As a consequence, ongoing clinical trials evaluating novel agents and combination regimens are aimed at personalizing CRC management [164].

In this context, the future directions include the assessment of emerging targeted therapies, such as NTRK, RAS, and HER2 inhibitors, as well as combination strategies, adding ICIs to the cytotoxic T-lymphocyte-associated protein 4 (CTLA-4) blockade to enhance therapeutic efficacy [164,165].

As a considerable subset of RC patients exhibits minimal or no response to nCRT, therapeutic resistance is considered to be a key factor that is associated with a poor clinical outcome [166,167]. Chemoradioresistance is driven by a complex molecular mechanism, including enhanced drug efflux, altered drug metabolism, reduced intracellular drug accumulation, and upregulated DNA repair pathways [167].

ANXA1’s high expression in pre-therapeutic biopsies has been strongly correlated with limited tumor regression following nCRT and with poor survival in LARC patients. Furthermore, ANXA1 is considered a biomarker of RC chemoradiotherapy resistance [27]. The mechanisms of ANXA1’s involvement in radioresistance are associated with TME modulation, interference with cellular stress response mechanisms, or involvement of the p53 signaling pathway [27]. Considering that ANXA1 acts as a context-dependent regulator of radiosensitivity, a potential use as both a predictive biomarker of radiotherapy response and a therapeutic target in treatment resistance prevention may be advocated in LARC [27].

Regarding the current treatment strategies, the neoadjuvant therapy represents a cornerstone of multimodal management, with two standard preoperative regimens widely employed in LARC: long-course chemoradiotherapy (LCRT), delivering 45–50 Gy in 25–28 fractions combined with fluoropyrimidine-based chemotherapy (5-FU or capecitabine), and short-course radiotherapy (SCRT), consisting of 25 Gy administered in five daily fractions [168,169,170]. The desired approach is LCRT, contributing to local control and tumor downstaging, while SCRT is chosen as an alternative in frail patients due to higher tolerability and reduced toxicity [168,169,171]. A major advancement in RC therapy is the introduction of total neoadjuvant therapy (TNT), which combines both systemic chemotherapy and chemoradiotherapy before the surgical intervention [169,172,173]. This approach aims to enhance treatment compliance, improve tumor regression, and optimize patients’ quality of life by reducing the period of therapy and by facilitating an early stoma closure [171,174], with an improved DFS and decreased rates of distant metastases by early targeting of micrometastatic disease [171,173,174].

Although different studies have explored the role of CRC and RC chemoradiotherapy/therapy/nCRT resistance, the molecular basis of this resistance is only partially deciphered. In this context, a close correlation between ANXA1 expression and 5-FU, a cornerstone chemotherapeutic agent in FOLFOX and FOLFIRI regimens in CRC management, has been detected [22,175]. Moreover, while ANXA1 is markedly upregulated in 5-FU-resistant colon cancer cells, its suppression has been shown to restore the drug sensitivity [25].

Increased ANXA1 expression markedly enhances the survival of CRC cells exposed to 5-FU, with a simultaneous suppression of the proteins that may counteract this effect [26]. In this respect, evidences suggest that ANXA1 is involved in 5-FU resistance through the regulation of the protein kinase C/c-Jun N-terminal kinase/P-glycoprotein (PKC/JNK/P-gp) signaling pathway [26], suppressing apoptosis, thereby reducing chemotherapy efficacy [176,177]. Thus, increased ANXA1 expression in CRC activates the signaling cascades that promote the expression of P-gp, a transmembrane efflux pump, thereby diminishing the intracellular accumulation of 5-FU and reducing its therapeutic effectiveness [26].

ANXA1 expression may be induced by hypoxia in CRC cells, with a possible role in resistance to 5-FU [26,178,179]. Hypoxia, a TME hallmark, may contribute to chemoresistance development by inducing cellular stress responses [26], associated with cell cycle arrest, suppression of cellular proliferation, and altered protein expression, impairing the efficacy of chemotherapeutic agents [25]. Both hypoxia and exposure to the hypoxia-mimetic agent cobalt chloride (CoCl_2_) may increase ANXA1 expression and hypoxia-inducible factor 1-alpha (HIF-1α) in breast cancer models [86,180]. Considering HIF-1α’s role as a key mediator of hypoxic signaling, it can interact with TP53, promoting tumor progression [25,181]. In an analogous manner, hypoxia may increase both ANXA1 and HIF-1α expression in experimental CRC, suggesting a potential link between hypoxia-driven responses and chemoresistance [25]. Given that HIF-1α inhibition may reverse CRC drug resistance in CRC, future studies may certify its value as a viable therapeutic target to overcome 5-FU resistance [25,182].

**Figure 2:**
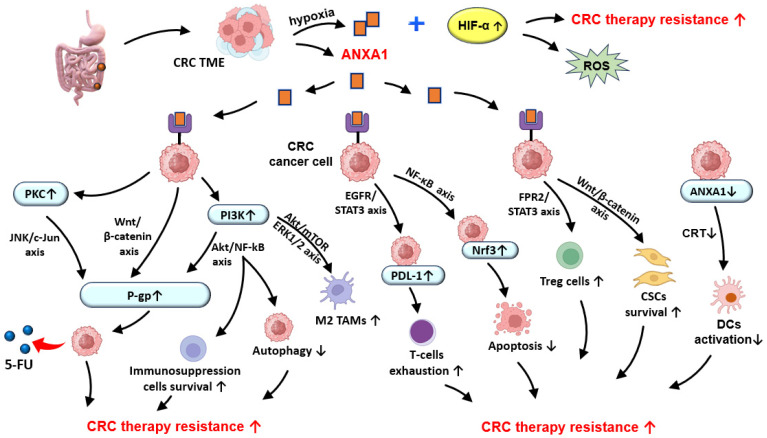
Potential role of ANXA1 in chemotherapy and radiotherapy resistance in CRC. ANXA1—Annexin A1; CRC—colorectal cancer; CRT—calreticulin; CSCs—cancer stem cells; DCs—dendritic cells; HIF-α—hypoxia-inducible factor 1-alpha; M2 TAMs—M2 phenotype tumor-associated macrophages; Nrf3—nuclear factor-erythroid 2-related factor 3; PDL-1—programmed death-ligand 1; P-gp—permeability glycoprotein; PKC—Protein kinase C; PI3K—phosphatidylinositol-3 kinase; ROS—reactive oxygen species; TAMs—tumor-associated macrophages; TME—tumor microenvironment; 5-FU—Fluorouracil; ↑—increase; ↓—decrease.

The phosphoinositide-3 kinase (PI3K)/Akt/mammalian target of rapamycin (mTOR) signaling axis (PI3K/Akt/mTOR) is an important regulator of tumor cells’ proliferation, growth, metabolism, and survival [183]. Dysregulation of this pathway, often driven by aberrant DNA methylation, has been identified as a key contributor to the development and progression of different malignancies, including colon cancer and RC [183,184]. Activation of the PI3K/Akt/mTOR pathway also modulates the CRC immune microenvironment by influencing cellular metabolism, promoting the survival of immunosuppressive cells, and enhancing the secretion of immunoregulatory cytokines and checkpoint molecules [185]. These immunosuppressive effects contribute to resistance against immune checkpoint inhibitors (ICIs), suggesting that their therapeutic targeting may reverse immunosuppression and enhance ICIs’ responsiveness [185]. Moreover, aberrant activation of this signaling cascade is closely associated with chemoresistance [183,184], with ANXA1 contributing to this process by autophagy inhibition via modulation of the PI3K/mTOR pathway [183,184]. In addition, ANXA1 promotes PD-L1 expression via EGFR/STAT3 pathway activation, supporting resistance to chemotherapy and immunotherapy by T-cell exhaustion and immune evasion [38,183,186]. A suppression of CD8^+^ cytotoxic T cells and an expansion of the Tregs population may also be induced by ANXA1 overexpression in TME by FPR2/STAT3 signaling activation, contributing to therapy resistance in several cancer types, including CRC [38,141] (Figure 2).

ANXA1 binds to macrophages’ FPR2, promoting TAMs polarization towards the immunosuppressive M2 phenotype in TME, by activating the PI3K/AKT and ERK1/2 pathways, leading to tumor progression stimulation by increasing cancer cell resistance to immune-mediated therapies [38,141,183]. Moreover, ANXA1 deficiency has been shown to facilitate immune escape in some cancers, including ANXA1-low CRC, by decreasing calreticulin exposure and dendritic cell activation, thus promoting the recruitment of cytotoxic T lymphocytes in TME and resistance to T-cell-induced chemotherapies, such as oxaliplatin [129,187].

The nuclear factor-kappa B (NF-κB) signaling pathway plays a pivotal role in cancer cell survival and inflammatory responses [188]. Its involvement has been extensively documented in different types of malignancies, including ovarian, breast, and prostate cancer, along with CRC [110]. NF-κB serves as a central regulator of CRC key oncogenic processes, such as angiogenesis, apoptosis, metastasis, inflammation, and chemoresistance [110,189]. Activation of NF-κB in response to chemotherapy has been associated with reduced therapeutic efficacy by the promotion of survival signals in tumor cells [110]. As a result, the combination of standard chemotherapeutic agents, such as 5-FU and oxaliplatin, with NF-κB inhibitors may enhance drug sensitivity, with an improvement in treatment outcomes [105]. For instance, co-treatment with NF-κB inhibitors, like SN50 or bortezomib, may suppress chemotherapy-induced NF-κB activation, thereby increasing cancer cell susceptibility to chemotherapeutic agents [105]. Moreover, evidence suggests that ANXA1 may also act as an upstream activator of NF-κB signaling, which activates nuclear factor-erythroid 2-related factor 3 (Nrf3), leading to decreased apoptosis and an increased resistance of CRC cells to 5-FU [25,74,190].

Other notable mechanisms of CRC chemoresistance are the Wnt ligand/β-catenin (Wnt/β-catenin) and PI3K/Akt/NF-κB signaling pathways [183,191]. Their aberrant activation supports malignant cell survival, promotes metastasis, and reduces therapy responsiveness [26]. Several studies have shown that ANXA1 plays a pivotal role in stimulating the Wnt/β-catenin [192,193] and PI3K/Akt/NF-κB pathways [191], followed by expression of the multidrug resistance protein 1 (MDR1) gene, encoding P-gp, thus contributing to CRC chemoresistance [191,192,193]. However, 5-FU in combination with flavonoids (curcumin) in lipid-based drug delivery can overcome P-gp-dependent efflux, leading to an increased 5-FU amount in CRC cells [166]. Additionally, the Wnt/β-catenin pathway plays a critical role in the regulation of cancer stem cell (CSC) properties, with a recognized role in CRC initiation, progression, and therapy resistance [26,194,195]. ANXA1 may also modulate the CSC population by supporting their maintenance and self-renewal capacities, contributing to tumor recurrence and reduced therapeutic efficacy [26,196].

Taken together, ANXA1 contributes to CRC chemoradioresistance by multiple interconnected mechanisms, such as the modulation of hypoxia-driven responses, activation of survival and drug efflux pathways, inhibition of autophagy, and enhancement of oncogenic signaling, such as PI3K/Akt/mTOR, NF-κB, and Wnt/β-catenin, providing a promising therapeutic target.

### 6.2. Perspectives of ANXA1 Exploitation in CRC-Targeted Therapy

Previous studies support that ANXA1 plays a major role in therapy resistance, showing a significant potential as a therapeutic target in different neoplasias. The therapeutic oncological potential of ANXA1 targeting has been suggested by its pivotal roles in immunomodulation and therapy resistance [26]. These findings have been explored using ANXA1 N-terminal mimetic peptide Ac2-26, which has shown promising value as an anticancer agent through the inhibition of cervical cancer cell proliferation [197] and modulation of the NF-κB signaling pathway in lung cancer [198].

Moreover, ANXA1 inhibition may enhance the anti-cancer efficacy of bortezomib—a key therapeutic agent in multiple myeloma (MM)—in both in vivo and in vitro models [199]. Furthermore, ANXA1 may represent a potential therapeutic target in non-small cell lung cancer, considering that its silencing leads to suppression of tumor cell proliferation, invasion, and migration [199]. Moreover, ANXA1 inhibition reduces Treg-mediated immunosuppression in TNBC. Furthermore, Ribonucleotide Reductase M2 (RRM2) stabilizes ANXA1 and modulates the therapeutic efficacy of PD-1 inhibitors and Sunitinib in renal cancer [68,200]. Additionally, ANXA1 targeting could be useful, considering that it promotes the progression and metastasis of nasopharyngeal carcinoma through the stabilization of ephrin type-A receptor 2 (EphA2) [89], and that exosomal ANXA1 is driving the malignant transformation of thyroid cells in thyroid cancer [32].

Another important ANXA1 characteristic is its ability to regulate the epithelial–mesenchymal transition (EMT)—a process that can either stimulate or inhibit tumor invasion [22]. ANXA1 involvement in cancer progression and metastasis is achieved by EMT initiation via multiple signaling cascades, e.g., PI3K/AKT, Wnt, TGF-β, MAPK/ERK, Notch, JNK, and p38 MAPK [22]. ANXA1 activation of the PI3K/AKT pathway, a well-established oncogenic axis [22,201], promotes CRC cell growth, invasion, and metastatic spread [22,201]. In the context of ANXA1’s role as a promising therapeutic target, a deeper insight into its role in EMT regulation may open the perspectives of innovative treatment strategies in CRC and other malignancies [22].

Emerging evidence suggests that ANXA1 may contribute to immune suppression within TME via its functional interaction with the EGFR pathway, a key regulator of cell proliferation and tumor progression in both colon cancer and RC [38,202]. The nuclear EGFR plays a co-transcriptional regulatory role that promotes treatment resistance and tumor aggressiveness [203]. In this context, ANXA1 facilitates EGFR activation and stabilization, supporting the development of new therapeutic targets in colon cancer and RC [42].

Punicalagin, a polyphenolic compound originating from pomegranate, is known for its antioxidant, anti-inflammatory, and anti-cancer effects, mainly in CRC [204]. Ganesan et al. conducted a proteomic analysis in HCT116 CRC cells to investigate the effect of punicalagin on proteins associated with apoptosis and focused on the ANXA1 signaling pathway [204]. This study evaluated the expression of 35 proteins and identified four proteins that showed significant alterations in cells treated with punicalagin and FPR inhibitors that are known to inhibit ANXA1 [204]. The analysis revealed that HSP27 and HSP60 proteins, along with tumor necrosis factor receptor 1 (TNFRI), were markedly downregulated, while the catalase value was increased [204]. Considering that HSP27 and HSP60 are generally involved in autophagy regulation and that TNFRI plays a central role in apoptosis, these findings suggest that punicalagin may induce CRC cells’ apoptosis by simultaneously modulating autophagic and apoptotic mechanisms by ANXA1 inhibition [204]. Therefore, the anticancerous properties of punicalagin in the HCT116 CRC cell line may be achieved through modulating the balance between apoptosis and autophagy via ANXA1 downregulation [204]. This is valuable information, considering that ANXA1 is often overexpressed in chemoresistant CRC, which is involved in cell survival and immune escape [22,25]. Moreover, natural agents, i.e., punicalagin, revealed therapeutic potential by inhibiting ANXA1 and re-establishing apoptosis–autophagy balance, suggesting that ANXA1 may be a future target in CRC therapy. In the same regard, another recent research revealed that granatin B and punicalagin showed potent anti-CRC and anti-mucositis activities in both HT-29 human CRC cell cultures and xenograft tumor models [205]. These effects occur via modulation of ROS-mediated S-phase cell cycle arrest and apoptosis in HT-29 cells [205]. Additionally, they enhance the sensitivity of HT-29 cells to 5-FU-induced cell death and S-phase arrest, suggesting that they could serve as potential new therapeutic agents in these oncologic patients [205].

Additionally, in a recent study, Al-Ali et al. investigated MDX-124, a novel humanized antibody directed against ANXA1, and demonstrated its ability to suppress the growth of ANXA1-expressing tumor cells both in vitro and in vivo by blocking its interaction with FPR1/2 [24]. According to this study, a 72 h treatment with MDX-124 reduced cancer cells’ viability in CRC, pancreatic, breast, and ovarian cancer cell lines, with a decreased metabolic activity, and, furthermore, induced G1-phase cell cycle arrest, thereby inhibiting proliferation [24].

There are limited clinical trials that explore the clinical efficacy of ANXA1-targeted therapy in oncologic patients. In this context, the phase Ib First-in-Human trial (ATTAINMENT) is currently underway to evaluate the safety, as well as the optimum dose of MDX-124, in a single administration or in combination with conventional oncologic therapy in adult patients with locally advanced, unresectable, or metastatic cancers [206]. The first module of this clinical trial started in August 2023, and, currently, the cohorts at 1, 2.5, and 5 mg/kg have been completed without dose-limiting toxicity. Meanwhile, enrolment for the 10 mg/kg MDX-124 cohort began in January 2024. MDX-124 also significantly impaired the migratory behavior of osteosarcoma cell lines and enhanced the efficacy of multiple standard-of-care therapies for osteosarcoma [207]. In this direction, a phase Ib clinical trial, which aims to explore the therapeutic potential of MDX-124 in pediatric osteosarcoma, is set to begin at the end of 2025 [207].

Our study had some limitations, which are mainly attributed to its retrospective design and reliance on computational approaches, such as bioinformatics analyses and modeling, which may be influenced by variability in patient demographics, tumor stage, and treatment regimens. The variability in cell lines, culture conditions, and experimental protocols across different studies may have also contributed to discrepancies in the results. This variability not only makes the direct comparisons between studies more complex, but it may also obscure underlying ANXA1 biological activities, highlighting the need for standardized approaches to increase the reproducibility and interpretability of the study’s findings. Moreover, the correlations observed between ANXA1 expression and immune cell infiltration are derived from experimental studies and lack the necessary validation by immunohistochemical methods or functional assays. To validate the preliminary studies, future investigations on large CRC patient cohorts are required. Additionally, elucidation of the downstream ANXA1 effectors in treatment-resistant tumor cells and elucidation of its interactions with other pathways of therapy resistance could set the development of novel strategies to overcome CRC chemoresistance, which are mainly registered in locally advanced tumors.

Based on the compiled data, ANXA1 demonstrates potential as a diagnostic and prognostic biomarker for CRC, a disease marked by heterogeneity and limited therapeutic response [68].

## 7. Conclusions

ANXA1 exhibits multiple roles in cell physiology and is recognized as a multifunctional protein that is involved in tumorigenesis and resistance to tumor therapy. Although multiple studies support its role in chemoresistance, further investigations are essential to certify its prognostic significance in different malignancies and to establish its potential value as a biomarker of treatment response.

ANXA1 is consistently correlated to CRC and RC development, progression, metastasis, and variable therapy responses. Its overexpression is correlated with aggressive cancer phenotypes and a poor prognosis, as well as resistance to nCRT and 5-FU, thus highlighting its possible role as a predictive and prognostic factor.

ANXA1 mediates therapy resistance in CRC and RC by modulation of different pathways, such as PI3K/AKT/mTOR, PKC/JNK/P-gp, Wnt/β-catenin, and NF-κB, while promoting immune evasion, stem cell maintenance, and a significantly immunosuppressive TME by DC cell recruitment and macrophage polarization.

In summary, the results emphasize the significance of ANXA1 as a relevant biomarker and therapeutic objective, and further research is warranted to elucidate its mechanisms with the ultimate goal of developing anti-ANXA1 strategies to mitigate therapeutic resistance in CRC and RC.

## Figures and Tables

**Table 1:** ANXA1 roles in different types of cancer.

Cancer Type	Study Models	ANXA1 Expression	Roles and Clinical Signification	References
Breast cancer	4T1 breast cancer cell lines	−/+++	Inhibits cancer macrophages’ infiltration Decreases tumor growth	[85]
	ANXA1 blocker Boc1 administration in balb/c mice and in TNBC patient tissue samples	−/+	Influences Tregs function Decreases tumor growth	[41]
	MDA-MB-231 cell line and TNBC patient tissue samples	+	Enhances invasiveness and improves metastatic potential Poor patient outcomes	[86]
Lung cancer	A549, H1703, H1650, H460, H1975, and H157 tumor cell lines	+++	Promotes invasion and tumorigenesis	[87]
	Mice xenograft lung cancer		Reduces tumor response to Osimertinib	
OSCC	SCC-9 and Tca-8113 cell lines	+	Potential marker value Suppresses proliferation and invasion Reverses TGF-β1/EGF-induced EMT	[88]
NPC	NPC patient tissue samples	+++	Stimulates metastasis occurrence	[89]
	6–10B and 5–8F NPC cell lines			[76]
Pancreatic cancer	Human MIA PaCa-2 cells	+++	Maintains and promotes a malignant phenotype	[90]
Melanoma	M4Beu, SK-MEL-3, M3Dau, and A375 cells lines	−/+	Promotes invasion and metastases	[62]
Glioblastoma	Human glioblastoma cell lines U251 and U87	+++	Poor OS and PFS Promotes proliferation and invasion Associated with higher WHO grades	[91]
Gastric cancer	Patient tissue samples AGS and N87 cell lines	−/+	Increased peritoneal metastasis risk Reduced OS	[92]
Prostate cancer	DU145, LNCaP, and PC3 cells lines	−	Enhances invasiveness and improves metastatic potential	[93]

ANXA1—Annexin A1; EGF—epidermal growth factor receptor; EMT—epithelial–mesenchymal transition; FPR2—Formyl peptide receptor 2; KO—knockout; NPC—nasopharyngeal carcinoma; OS—overall survival; OSCC—oral squamous cell carcinoma; PFS—progression-free survival; TGF-β1—Transforming growth factor-β1; TNBC—triple-negative breast cancer; WHO—World Health Organization. “–“—negative; “+’’—positive. “−/+”—variable. “+++”—intensely positive.

**Table 2:** ANXA1 expression pattern and its functional correlations in colon vs. rectal cancer.

Parameters	Study Model	Colon Cancer and CRC		RC		References
		ANXA1 Expression	Functional Correlation	ANXA1 Expression	Functional Correlation	
TNM status	Patients’ tissue samples	Positive	T3 and T4 stages	Positive	T3 and T4 stages	[17,21,27,94]
Lymph node metastasis	Patients’ tissue samples	Positive	Positive	Positive	N2 and N3 stages	[17,27,94]
Tumor angiogenesis and vascular invasion	Patients’ tissue samples	Positive	Vascular invasion	Positive	Vascular invasion	[17,27]
Metastasis	Patients’ tissue sample	Positive	Variable positive	Positive	MeFS ↓	[17,27]
Prognosis	Patients’ tissue samples	Positive	DSS ↓ OS ↓	Positive	DSS ↓ OS ↓ LRFR ↓	[17,27,68,128]
Serological marker	Patients’ serum samples	Value ↑ Value ↓	Positive diagnosis Systemic inflammation	-	-	[94,103]
Tumor cells’ cycle	HCT116 and SW620 colon carcinoma cell lines Patients’ tissue samples	Positive	tumor cells proliferation ↑ tumor cells apoptosis ↓	-	-	[25,68,94,112]
TME hypoxia	SW620, HCT116, and SW48 colon carcinoma cell lines	Positive	HIF-1α ↑	-	-	[25]
Tumor-infiltrating immune cells	Patients’ tissue samples	Positive	CD8^+^Ly and NK activity ↓ M2 TAMs infiltration ↑ Treg cells activity ↑ DCs differentiation ↓ Tumor immune infiltrate ↑	-	-	[128]
		Negative				[29]

ANXA1—Annexin 1; CRC—colorectal cancer; DCs—dendritic cells; DSS—disease-specific survival; HIF-1α—Hypoxia-inducible factor 1-alpha; LRFS—local recurrent-free survival; Ly—lymphocytes; MeFS—metastasis-free survival; NK—natural killer cell; OS—overall survival; RC—rectal cancer; TAMs—tumor-associated macrophages; Treg cells—T-regulatory cells; ↑—increase; ↓—decrease.

## Data Availability

No new data were created or analysed in this study.
